# Safety assessment of rosuvastatin-fenofibrate combination in the treatment of hyperlipidemia based on FDA’s adverse event reporting system database

**DOI:** 10.3389/fphar.2025.1415701

**Published:** 2025-02-06

**Authors:** Qun Li, Wenya Shan, Saiwei Wu

**Affiliations:** ^1^ Department of Pharmacy, The Fourth Affiliated Hospital of School of Medicine, International School of Medicine, International Institutes of Medicine, Zhejiang University, Yiwu, Zhejiang, China; ^2^ Department of Pharmacy, The First Affiliated Hospital of School of Medicine, Zhejiang University, Hangzhou, Zhejiang, China

**Keywords:** FAERS, rosuvastatin, fenofibrate, adverse events, pharmacovigilance

## Abstract

**Background:**

With the improvement of living standards, an increasing number of patients are presenting with mixed hyperlipidemia. In addition to cholesterol reduction, it is imperative to lower triglyceride levels. The combination of statin and fibrate for reducing lipid levels has commonly been applied in clinical therapy. However, the combination of drugs also increases the risk of adverse events (AEs). In this study, we analyzed the safety signals of rosuvastatin-fenofibrate combination by assessing the publicly available US Food and Drug Administration Adverse Event Reporting System (FAERS), so as to provide a reference for rational clinical use of rosuvastatin and fenofibrate, and reduce the occurrence of related AEs.

**Methods:**

Reports to the FAERS from 1 January 2004 to 19 March 2020 were analyzed. The proportional report ratio (PRR), reporting odds ratio (ROR), and Bayesian Confidence Propagation Neural Network (BCPNN) analysis were used to extract data from FAERS for suspected signals referring to the combination of rosuvastatin and fenofibrate.

**Results:**

A total of 68 safety signals were detected from the top 250 AEs in 3,587 reports, of which 28 signals were not included in the drug labels. All the detected AEs were associated with 12 System Organ Classes (SOC), such as gastrointestinal, musculoskeletal and connective tissue, general diseases, investigations and nervous system. The most frequent AEs were analyzed, and it was found that women generally have a higher susceptibility to experiencing AEs, including pain, nausea, fatigue, myalgia, diarrhea, dyspnea, headache, weakness, and dizziness.

**Conclusion:**

Clinicians should pay more attention to the AEs of gastrointestinal and muscular system during combination therapy, and it is recommended to strengthen pharmaceutical care during clinical application.

## Introduction

With the improvement of individuals’ living standards, there has been a significant increase in the prevalence of dyslipidemia. Genetic defects and unhealthy lifestyle are two risk factors of hyperlipidemia (Lorenzatti and Toth, 2020), especially, the change in people’s dietary structure towards high fat, high sugar and high calorific value, as well as ultra-processed foods, has led to a sharp increase in the prevalence of dyslipidemia (Arnett et al., 2019; Juul et al., 2021). Hypercholesterolemia stands as the primary contributor to cardiovascular diseases, which is one of the main reasons for adult death in the United States and causes huge economic losses every year (Dawber et al., 2015; Virani et al., 2020). Numerous guidelines advocate for statin usage to mitigate the morbidity and mortality associated with such conditions (Lloyd-Jones et al., 2017; Mach et al., 2020; Stone et al., 2014). Low density lipoprotein cholesterol (LDL-C) serves as a pivotal target for intervention in reducing atherosclerotic cardiovascular disease (ASCVD). Patients with hypertriglyceridemia exhibit elevated levels of residual lipoproteins that are likely to exert atherogenic effects. Consequently, non-high-density lipoprotein cholesterol (HDL-C) is also employed as an auxiliary intervention target. Although statins effectively lower LDL-C levels, the achievement of comprehensive lipid regulation necessitates the concomitant use of other lipid-modulating agents.

3- Hydroxy-3-methylglutaryl-coenzyme A (HMG-CoA) reductase inhibitors (statins) have been widely recommended to reduce the incidence rate and mortality of cardiovascular diseases (Heart Protection Study Collaborative Group, 2002; Lloyd-Jones et al., 2017; Mach et al., 2020; Pedersen et al., 2004; Stone et al., 2014). Statins primarily function by inhibiting HMG-CoA reductase, thereby impeding cholesterol synthesis within the body. Fibrates can augment lipoprotein lipase activity and diminish triglyceride levels. The 2019 ESC Lipid Guidelines suggest that a combination of statins and fibrates may be considered when a patient’s TG > 2.3 mmol/L (Mach et al., 2020). According to the 2023 Chinese Lipid Management Guidelines, individuals with ASCVD or at high risk should receive moderate dose statin therapy if their TG > 2.3 mmol/L, and fibrates can be administered to further mitigate the risk of ASCVD (Joint Committee on the Chinese Guidelines for Lipid Management, 2023). Even so, the cardiovascular benefits of statins in combination with fibrates remain a subject of debate and controversy within the scientific community. The safety of combining statins and fibrates in the Chinese population is deemed acceptable; however, further verification is required to establish the long-term safety of this combination (Joint Committee on the Chinese Guidelines for Lipid Management, 2023). Nevertheless, due to the similar metabolic pathways of statins and fibrates, their combination has the potential to cause liver injury and increase the risk of myositis and myopathy (Joint committee for guideline, 2018), greatly increasing the occurrence rate of adverse events (AEs). It is well-established that the concurrent administration of statins and fibrates can give rise to significant adverse reactions. In 2001, cerivastatin, a promising statin, was introduced to the market; however, Bayer Pharmaceutical, its manufacturer, subsequently contraindicated the combination of cerivastatin and gemfibrozil due to frequent and severe reports of rhabdomyolysis-related deaths (Staffa et al., 2002). Consequently, Bayer withdrew cerivastatin from the international drug market that same year (Wooltorton, 2001). Overall, the drug labels of AEs after the combination therapy of these two drugs is still deficient, which is not conducive to actual clinical applications. The safety of combining statins and fibrates should be given significant attention.

The real-world data could provide post-marketing drug safety information, which is beneficial for clinicians to weigh risks and benefits. The US Food and Drug Adiministration (via the FDA Adverse Event Reporting System, FAERS), the World Health Organisation (via VigiBase) and the European Medicines Agency (via Eudra Vigilance) are the most widely used databases for reporting spontaneous adverse drug reactions abroad. FAERS Data files are provided in ASCII or SGML format to ensure consistency in compiling drug and adverse event data. Information transfer between databases is carried out directly using standardized data formats, as FDA only accepts electronic submissions of ICSRs in XML format. Herein, this study is aimed to analyze the AEs reports of FAERS, so as to provide references for rational clinical application through detecting safety signals and identifying potential drug risk signals.

## Methods

### Data source

In this study, we obtained data from the OpenFDA, a public data open project in the United States, and the original data of AEs were imported by FAERS (Joint committee for guideline, 2018). FAERS collects spontaneous safety reports and post-marketing clinical research reports related to drugs used in the United States and abroad. All AEs were coded using preferred terms (PT) from the Medical Dictionary for Regulatory Activities (MedDRA) (Ma et al., 2021).

We used Research AE as the analysis tool to extract AEs reports from the FAERS database which covered the period from 1 January 2004 to 19 March 2020. Research AE is a research AE analysis tool, which can directly extract AEs from the FAERS database through the interface of application programming (API). The generic names of rosuvastatin and fenofibrate were used as the keywords to perform searches, and the AEs reports were included when rosuvastatin and fenofibrate were the first suspect drugs. Reports pertaining to diseases, which related to drug indications, or concomitant disease were excluded from the analysis, other reports from the top 250 AE cases were left for signal detection in order to assess the association between drugs and AEs.

### Signal detection method

Disproportionality analysis is a commonly used analytic method for AEs signal mining, which could be divided into two categories: frequentist and Bayesian methods. No “gold standard” is available, each of the above methods has its own shortages (van Puijenbroek et al., 2003). Both proportional reporting ratio (PRR) (Evans et al., 2001) and reporting odds ratio (ROR) (van Puijenbroek et al., 2002) are frequency methods. They are easy for calculation and can lead to a more sensitive output than bayesian approaches. The bayesian confidence propagation neural network (BCPNN) (Noguchi et al., 2019) is always applicable and large numbers of calculations can be made efficiently. Both approaches entailed inherent drawbacks, including: the limitation of frequentist statistical method mainly includes: i): false positive signals might be detected and ii): measured values are sensitive to small fluctuations. Correspondingly, the restriction of Bayesian Confidence Propagation Neural Network (BCPNN) mainly including: i): false-negative signals might be detected. ii): measured values are not specific and iii): signal value is difficult to be calculated (Bate et al., 2002; Noguchi et al., 2021). No one algorithm is universally better than others. In the present investigation, we used PRR, ROR, and BCPNN for safety signals detection. The two-by-two frequency table of disproportionality analysis is shown in Table 1.

**TABLE 1:** Two-by-two frequency table.

Project	Adverse event of interest	All other adverse events	Total
Drug of interest	a	b	a + b
All other drugs	c	d	c + d
Total	a + c	b + d	a + b + c + d

a, the incidence of specific adverse events attributed to the drug. b, the incidence of non-specific adverse events associated with the drug. c, the incidence of specific adverse events reported for all drugs, excluding the drug in question. d, the incidence of non-specific adverse events reported for all drugs, excluding the drug under investigation.

Herein, the criteria of PRR and ROR were: a ≥ 3, the lower bound of 95% two-sided confidence interval (CI) > 1, and the criteria of BCPNN were: IC-2SD > 0 (Shen et al., 2019) and the algorithm was showed in Equation 1. The higher the scores of PRR, ROR, and BCPNN, the stronger the association between the drugs and AEs. In addition, to identify the impact of gender differences on AEs, we analyzed 10 AEs most frequently reported and performed ROR analysis (ROR > 1 means a higher likelihood of AEs occurring in females).

$$\begin{array}{c} \alpha_{1}=\beta_{1}=1;\;\alpha =\beta =2;\;\gamma_{11}=1;\;C=a+b+c+d;\;C_{x}=a+b;\;C_{y}=a+c;\;C_{xy}=a;\\ \gamma =\gamma_{11}\frac{\left(C+\alpha \right)\left(C+\beta \right)}{\left(C_{x}+\alpha_{1}\right)\left(C_{y}+\beta_{1}\right)};\;E\left(IC\right)=\log_{2}\frac{\left(C_{xy}+\gamma_{11}\right)\left(C+\alpha \right)\left(C+\beta \right)}{\left(C+\gamma \right)\left(C_{x}+\alpha_{1}\right)\left(C_{y}+\beta_{1}\right)};\\ V\left(IC\right)=\frac{1}{{\left(\ln2\right)}^{2}}\left\{\left(\frac{C-C_{xy}+\gamma -\gamma_{11}}{\left(C+\gamma_{11}\right)\left(1+C+\gamma \right)}\right)+\left(\frac{C-C_{x}+\alpha -\alpha_{1}}{\left(C_{x}+\alpha_{1}\right)\left(1+C+\alpha \right)}\right)+\left(\frac{C-C_{y}+\beta +\beta_{1}}{\left(C_{y}+\beta_{1}\right)\left(1+C+\beta \right)}\right)\right\};\\ IC-2SD=E\left(IC\right)-2\sqrt{V\left(IC\right)} \end{array}$$ (1)

## Results

### AEs reports and demographic characteristics of patients

In this study, a total of 3,587 AEs were reported with rosuvastatin and fenofibrate as the first suspect drugs. As shown in Table 2, doctors (21.60%), pharmacists (6.08%), and other medical staff (14.75%) were main reporters. The highest proportion of reports was by consumers and non-medical staff. In addition, there are more male patients (54.47%) than female patients (41.43%) and the patients aged 45∼64 are counted the most percent (31.28%). Notably, the percentage of serious AEs was 43.41% after combined therapy, of which 1,110 (30.95%) reported cases were hospitalization or prolonged hospitalization.

**TABLE 2:** Demographic characteristics of patients and composition of serious adverse events.

Variables	Reports (n)	Percentage (%)
Gender		
Male	1954	54.47
Female	1,486	41.43
Unknown	147	4.10
Age, years		
<18	6	0.17
18–44	157	4.38
45–64	1,122	31.28
65–74	587	16.36
≥75	299	8.34
Unknown	1,416	39.48
Occupation		
Doctor	775	21.60
Pharmacist	218	6.08
Other medical staff	529	14.75
Lawyer	67	1.87
Consumer or non-medical Staff	1,386	38.64
Unknown	612	17.06
Serious adverse events		
Death	202	5.63
Hospitalization	1,110	30.95
Congenital anomalies	1	0.03
Disabling	121	3.37
Life threatening	123	3.43

### Signal detection of AEs

As defined in MedDRA, the safety signals were classified according to System Organ Class (SOC). Herein, a total of 68 safety signals were detected from the top 250 AEs in 3,587 events. As shown in Tables 3 40 (58.82%) safety signals, involving 12 SOC, were listed in the drug labels, of which the top 5 AEs were gastrointestinal diseases (468 reports, 13.05%), musculoskeletal and connective tissue diseases (400 reports, 11.15%), general diseases (292 reports, 18.14%), investigations (272 reports, 7.58%) and nervous system diseases (186 reports, 5.19%), respectively. In addition, 28 (41.18%) signals were not included in the drug labels, which mainly including blushing, back pain, weight loss, poor appetite and so on.

**TABLE 3:** Significant disproportionality results displayed according to SOC and PT.

PT	Reports	Percentage (%)	PRR (95% CI)	ROR (95% CI)	IC (IC-2SD)	Listed in the drug labels
Blood and lymphatic system disorders						
Anemia	30	0.84%	1.57 (1.10, 2.24)	1.57 (1.10, 2.26)	0.62 (0.09)	No
Nephrogenic anemia	21	0.59%	48.35 (31.55, 74.10)	48.72 (31.64, 75.04)	3.93 (3.31)	No
Hemorrhagic anemia	7	0.20%	6.20 (2.96, 13.00)	6.21 (2.95, 13.04)	1.91 (0.88)	No
Gastrointestinal disorders						
Nausea	112	3.12%	1.52 (1.27, 1.82)	1.52 (1.26, 1.84)	0.57 (0.28)	Yes
Diarrhea	92	2.56%	1.70 (1.40, 2.08)	1.70 (1.38, 2.10)	0.73 (0.41)	Yes
Constipation	47	1.31%	2.60 (1.96, 3.44)	2.60 (1.94, 3.47)	1.31 (0.88)	Yes
Abdominal pain	34	0.95%	1.54 (1.10, 2.15)	1.54 (1.10, 2.16)	0.59 (0.09)	Yes
Gastrointestinal bleeding	34	0.95%	3.27 (2.35, 4.57)	3.28 (2.33, 4.60)	1.60 (1.11)	No
Epigastric pain	31	0.86%	1.64 (1.16, 2.33)	1.64 (1.15, 2.34)	0.68 (0.16)	Yes
Gastroesophageal reflux disease	25	0.70%	3.28 (2.22, 4.84)	3.28 (2.21, 4.87)	1.58 (1.01)	No
Abdominal discomfort	22	0.61%	1.60 (1.06, 2.42)	1.60 (1.05, 2.44)	0.63 (0.03)	Yes
Indigestion	21	0.59%	2.26 (1.48, 3.46)	2.26 (1.47, 3.48)	1.09 (0.47)	Yes
Pancreatitis	17	0.47%	3.00 (1.87, 4.82)	3.01 (1.86, 4.85)	1.43 (0.74)	Yes
Gastrointestinal disorder	14	0.39%	2.22 (1.31, 3.73)	2.22 (1.31, 3.75)	1.03 (0.28)	Yes
Upper gastrointestinal bleeding	10	0.28%	5.04 (2.71, 9.35)	5.04 (2.71, 9.39)	1.88 (1.00)	No
Acute pancreatitis	9	0.25%	3.76 (1.96, 7.21)	3.76 (1.95, 7.24)	1.55 (0.64)	Yes
General disorders and administration site conditions						
Fatigue	131	3.65%	2.01 (1.70, 2.37)	2.01 (1.68, 2.01)	0.94 (0.68)	Yes
Powerless	71	1.98%	2.00 (1.59, 2.52)	2.00 (1.58, 2.00)	0.95 (0.60)	Yes
Pain	69	1.92%	1.39 (1.10, 1.75)	1.39 (1.09, 1.77)	0.45 (0.09)	No
Edema	11	0.31%	2.06 (1.14, 3.71)	2.06 (1.14, 3.72)	0.91 (0.08)	Yes
Hearing loss	10	0.28%	3.29 (1.77, 6.10)	3.29 (1.77, 6.13)	1.44 (0.56)	No
Investigations						
Weight loss	55	1.53%	2.18 (1.68, 2.83)	2.18 (1.67, 2.85)	1.07 (0.68)	No
Increased blood glucose concentration	54	1.51%	2.69 (2.06, 3.49)	2.69 (2.05, 3.52)	1.36 (0.95)	Yes
Elevated serum creatinine phosphokinase	28	0.78%	7.87 (5.45, 11.36)	7.88 (5.42, 11.44)	2.65 (2.11)	Yes
Increased blood creatinine	27	0.75%	4.11 (2.83, 5.98)	4.11 (2.81, 6.02)	1.87 (1.32)	Yes
Decreased white blood cell count	19	0.53%	2.05 (1.31, 3.21)	2.05 (1.30, 3.22)	0.95 (0.30)	Yes
Abnormal liver function	17	0.47%	4.44 (2.77, 7.13)	4.45 (2.76, 7.17)	1.89 (1.20)	Yes
Increased glycosylated hemoglobin	16	0.45%	7.20 (4.42, 11.73)	7.21 (4.40, 11.79)	2.39 (1.69)	Yes
Increased aspartate aminotransferase	14	0.39%	2.43 (1.44, 4.09)	2.43 (1.44, 4.11)	1.14 (0.39)	Yes
Increased alanine aminotransferase	13	0.36%	1.99 (1.16, 3.42)	1.99 (1.15, 3.43)	0.89 (0.11)	Yes
Increased liver enzymes	12	0.33%	2.17 (1.23, 3.81)	2.17 (1.23, 3.82)	0.99 (0.18)	Yes
International standardization ratio rises	9	0.25%	2.20 (1.15, 4.23)	2.20 (1.14, 4.24)	0.97 (0.05)	No
Elevated prostate specific antigen	8	0.22%	6.16 (3.08, 12.31)	6.17 (3.08, 12.35)	1.97 (1.00)	No
Metabolism and nutrition disorders						
Diabetes	41	1.14%	5.21 (3.85, 7.05)	5.21 (3.82, 7.10)	2.22 (1.76)	Yes
Poor appetite	30	0.84%	1.50 (1.05, 2.14)	1.50 (1.05, 2.15)	0.55 (0.03)	No
Dehydration	24	0.67%	1.74 (1.17, 2.59)	1.74 (1.16, 2.60)	0.75 (0.16)	No
Musculoskeletal and connective tissue disorders						
Myalgia	87	2.43%	5.23 (4.26, 6.42)	5.23 (4.22, 6.49)	2.26 (1.94)	Yes
Limb pain	55	1.53%	1.89 (1.46, 2.45)	1.89 (1.44, 2.47)	0.87 (0.48)	No
Joint pain	50	1.39%	1.47 (1.12, 1.93)	1.47 (1.11, 1.94)	0.53 (0.11)	Yes
Backache	40	1.12%	1.80 (1.32, 2.44)	1.80 (1.31, 2.46)	0.80 (0.34)	No
Muscle cramps	34	0.95%	1.85 (1.33, 2.59)	1.85 (1.32, 2.60)	0.84 (0.35)	Yes
Rhabdomyolysis	31	0.86%	7.24 (5.10, 10.26)	7.25 (5.08, 10.34)	2.58 (2.06)	Yes
Myasthenia	27	0.75%	2.38 (1.64, 3.46)	2.38 (1.63, 3.48)	1.17 (0.62)	Yes
Muscular atrophy	23	0.64%	18.09 (12.04, 27.16)	18.14 (12.01, 27.38)	3.39 (2.79)	No
Myopathy	17	0.47%	16.01 (9.97, 25.71)	16.04 (9.95, 25.88)	3.12 (2.43)	Yes
Muscle fatigue	14	0.39%	3.35 (1.99, 5.65)	3.35 (1.98, 5.67)	1.53 (0.78)	No
Intervertebral disc degeneration	11	0.31%	10.83 (6.01, 19.53)	10.85 (5.99, 19.63)	2.57 (1.73)	No
Osteoarthritis	11	0.31%	2.44 (1.35, 4.39)	2.44 (1.35,4.41)	1.12 (0.28)	No
Nervous system disorders						
Headache	87	2.43%	1.47 (1.20, 1.81)	1.47 (1.19, 1.83)	0.53 (0.21)	Yes
Dizzy	66	1.84%	1.37 (1.08, 1.73)	1.37 (1.07, 1.75)	0.43 (0.06)	Yes
Syncope	19	0.53%	1.87 (1.20, 2.93)	1.87 (1.19, 2.94)	0.83 (0.18)	No
Diabetic neuropathy	14	0.39%	20.68 (12.26, 34.88)	20.75 (12.25, 35.13)	3.16 (2.41)	No
Psychiatric disorders						
Insomnia	45	1.25%	1.66 (1.24, 2.22)	1.66 (1.24, 2.23)	0.70 (0.26)	Yes
Sleep disorders	24	0.67%	4.35 (2.92, 6.47)	4.35 (2.91, 6.51)	1.93 (1.34)	Yes
Irritability	13	0.36%	1.91 (1.11, 3.28)	1.91 (1.11, 3.30)	0.84 (0.06)	No
Abnormal dream	10	0.28%	2.89 (1.56, 5.36)	2.89 (1.55, 5.38)	1.30 (0.42)	Yes
Renal and urinary disorders						
Renal damage	28	0.78%	4.05 (2.81, 5.86)	4.06 (2.79, 5.89)	1.86 (1.32)	Yes
Renal calculus	20	0.56%	5.19 (3.36, 8.03)	5.19 (3.34, 8.07)	2.10 (1.47)	No
Hematuria	11	0.31%	2.97 (1.64, 4.96)	2.97 (1.64, 5.37)	1.34 (0.51)	Yes
Dysuria	10	0.28%	2.67 (1.44, 4.96)	2.67 (1.43, 4.97)	1.21 (0.33)	No
Respiratory, thoracic and mediastinal disorders						
Dyspnea	69	1.92%	1.31 (1.04, 1.65)	1.31 (1.03, 1.66)	0.37 (0.01)	Yes
Chronic obstructive pulmonary disease	12	0.33%	2.27 (1.29, 4.00)	2.27 (1.29,4.01)	1.04 (0.24)	No
Skin and subcutaneous tissue disorders						
Itch	49	1.37%	1.70 (1.29, 2.24)	1.70 (1.28, 2.26)	0.73 (0.31)	Yes
Skin damage	9	0.25%	3.38 (1.76, 6.49)	3.39 (1.76, 6.52)	1.45 (0.53)	Yes
Eczema	8	0.22%	3.65 (1.82, 7.29)	3.65 (1.82, 7.31)	1.49 (0.52)	No
Vascular disorders						
Blush	42	1.17%	3.71 (2.75, 5.01)	3.71 (2.73, 5.04)	1.78 (1.33)	No
Hemorrhage	19	0.53%	1.91 (1.22, 2.99)	1.91 (1.22, 3.01)	0.87 (0.21)	No
Thromboembolism	14	0.39%	1.85 (1.10, 3.12)	1.85 (1.09, 3.13)	0.80 (0.05)	Yes

Furthermore, according to the analysis of AEs in Table 4, we found that females exhibit a higher susceptibility to experiencing AEs, including pain, nausea, fatigue, myalgia, diarrhea, dyspnea, headache, weakness, and dizziness. Correspondingly, men are more likely to experience weight loss.

**TABLE 4:** Gender differences in adverse event reactions.

PT	Reports	ROR (95%CI)
Pain	252	1.21 (1.00, 1.45)
Nausea	115	1.62 (1.23, 2.15)
Fatigue	92	1.04 (0.78, 1.37)
Myalgia	91	1.43 (1.06, 1.94)
Diarrhea	83	1.48 (1.08, 2.04)
Dyspnea	80	1.68 (1.20, 2.35)
Headache	73	1.37 (0.98, 1.91)
Powerless	66	1.06 (0.76, 1.48)
Dizzy	61	1.12 (0.79, 1.58)
Weight loss	49	0.87 (0.60, 1.25)

ROR > 1 indicates females are more likely to have AEs, ROR < 1 indicates that males are more likely to have AEs.

## Discussion

According to the signal screening results, during the combined treatment of rosuvastatin and fenofibrate, the risks of gastrointestinal system disorders, musculoskeletal and connective tissue disorders, general disorders, medical tests as well as neurological disorders were increased when rosuvastatin and fenofibrate were applied in combination, which were consistent with previous researches (Ferdinand et al., 2012; Pepine et al., 2010; Roth et al., 2010). And the most commonly reported AEs were in the gastrointestinal system, mainly manifested as nausea, diarrhea and other discomfort, which would affect patients’ appetite and sleep quality, thus further increasing their discomfort and even cause discontinuation of treatment in severe cases, greatly limits the therapeutic effect of patients. To improve patients’ medication compliance, medication guide and health education could be strengthened, enabling them fully understand disease and drugs, reducing psychological burden and adjusting the diet as needed.

In addition, since both statins and fibrates have the potential to cause liver injury, myositis and myopathy, their combination is more likely to cause liver and kidney damage as well as muscle aches (Cranmer et al., 2021; Shen et al., 2019; van Puijenbroek et al., 2002). Therefore, it is recommended to closely monitor the indices of creatine kinase and liver enzymes, as well as reporting all unexplained muscle aches and pains. Besides, for special populations, such as the elderly and children, the overweight or slim people and patients simultaneously using several drugs, the dose could be adjusted according to the patient’s tolerance to avoid serious AEs (Alomar, 2014; Han et al., 2022; Zhang et al., 2023).

Furthermore, basing on the signal screening of FAERS database, we found that 28 signals were not included in the drug label, mainly including flushing, back pain, weight loss and loss of appetite, which suggests possible AEs outside instructions during the actual application of rosuvastatin and fenofibrate. Hence, our research is expected to provide data support beyond the instructions for rapid clinical evaluation of combined drugs.

However, this study still remains some deficiencies. On account of the detection of signal was based on the spontaneous reporting database, it was prone to have missed, duplicate, incomplete and irregular reports. While consumers or non-medical staff reports constituted the largest proportion, this subset of reports showed a greater tendency for incompleteness and irregularity, which consequently affected the accuracy of data analysis. In addition, the disproportionality analysis was focused on the number of reports, which failed to take the time-to-onset distribution into account (Noguchi et al., 2021). It also did not take into account patients’ basic diseases and other combined medication issues as well as reports that one drug was regarded as a suspicious drug and another drug was regarded as an accompanying drug. Besides, because it is difficult to identify which patient was prescribed with these drugs and for what reason, many heterogeneous patients were also included in our analyzation. Additionally, since PT was fixed, we counted the AEs and checked them with the drug labels objectively, which might bias the judgment of whether the adverse event was expected or not. Moreover, the safety signals detected in the study only indicated a statistical correlation between drugs and AEs, specific methods to investigate drug-drug interaction are still need to be considered in further studies.

## Conclusion

Based on the FDA adverse event database, this study identified a total of 68 positive signals. When rosuvastatin was combined with fenofibrate, the most prevalent AEs observed were related to gastrointestinal system diseases, musculoskeletal and connective tissue diseases, general diseases, investigations and nervous system diseases. Additionally, analysis of FAERS database data revealed 28 signals primarily associated with blushing, back pain, weight loss, poor appetite and so on, were not included in the drug labels. Therefore, more attention needs to be paid to the combined therapy of statin and fibrate. And we believe our real-world data analysis could be expected to provide helpful reference for rapid clinical assessment and further promote rational clinical medication.

## Data Availability

The datasets presented in this study can be found in online repositories. The names of the repository/repositories and accession number(s) can be found in the article/supplementary material.
